# Development of 3D Printed Drug-Eluting Scaffolds for Preventing Piercing Infection

**DOI:** 10.3390/pharmaceutics12090901

**Published:** 2020-09-22

**Authors:** Emad Naseri, Christopher Cartmell, Matthew Saab, Russell G. Kerr, Ali Ahmadi

**Affiliations:** 1 Faculty of Sustainable Design Engineering, University of Prince Edward Island, 550 University Avenue, Charlottetown, PE C1A 4P3, Canada; enaseri@upei.ca; 2 Department of Chemistry, University of Prince Edward Island, 550 University Avenue, Charlottetown, PE C1A 4P3, Canada; ccartmell@upei.ca (C.C.); rkerr@upei.ca (R.G.K.); 3 Atlantic Veterinary College, University of Prince Edward Island, 550 University Avenue, Charlottetown, PE C1A 4P3, Canada; msaab@upei.ca; 4 Nautilus Biosciences Croda, Regis and Joan Duffy Research Centre, 550 University Avenue, Charlottetown, PE C1A 4P3, Canada

**Keywords:** biopierce, 3D printing, PLGA, bacterial test, drug eluting scaffolds

## Abstract

Herein, novel drug-eluting, bio-absorbable scaffold intended to cover piercing studs is introduced. This “biopierce” will stay in human tissue following piercing, and will slowly release an antimicrobial agent to prevent infection while the wound heals. Nearly 20% of all piercings lead to local infection. Therefore, it is imperative to develop alternative methods of piercing aftercare to prevent infection. Biopierces were made using mupirocin loaded poly-lactic-*co*-glycolic acid (PLGA) biomaterial ink, and a low-temperature 3D printing technique was used to fabricate the biopierces. Proton nuclear magnetic resonance (^1^H NMR) spectroscopy was used to confirm the complete removal of the solvent, and liquid chromatography high-resolution mass spectrometry (LC-HRMS) was used to confirm the structural integrity of mupirocin and to quantify the amount of the released drug over time. The efficacy of the biopierces against *Staphylococcus aureus*, one of the most common piercing-site pathogens, was confirmed over two weeks using in vitro antimicrobial susceptibility testing.

## 1. Introduction

Body piercing has become very popular in the past two decades, and it has been reported that approximately 50% of millennials receive at least one non-earlobe piercing [1,2]. As most of these piercings are not performed in clinical settings, 17–46% of the piercings lead to complications [3,4] including infection, metal allergy, bleeding, and tissue scarring [4,5,6,7]. Among all these problems, local infection has been reported to be the most common type of piercing complication [6]. *Staphylococcus aureus* (*S. aureus*) and *Streptococcus pyogenes* (*S. pyogenes*) (Lancefield Group A) are the most common pathogens causing these local infections [6,8]. Particularly, as compared to the wounds stemming from the piercing of soft tissue, cartilage piercings (e.g., the auricle of the ear) have a greater predisposition to complications [9,10]. These complications are due to the detachment of perichondrium and microfractures of the cartilage during piercing, which consequently leads to edema and bleeding into the cartilage [10]. This trauma to the tissue ultimately results in a reduction of blood circulation to the relatively avascular cartilage tissue and increases the risk of infection.

Due to the avascular nature of cartilage, systemic antibiotic treatment through oral intake cannot be prescribed to patients with piercing infections in the auricle tissue, and the treatment for severe infections may require hospitalization and surgical intervention [11]. It has been reported that a treatment delay greater than five days for cartilage infections can lead to severe outcomes, such as impaired hearing, ear deformation, and amputation of the auricle [10]. There has been much effort to prevent infection resulting from wounds by administering a wide range of active agents to the wound locally: these agents include anti-infective [12,13,14], anti-inflammatory [15,16,17], and analgesic [18,19,20] compounds. The common aftercare practice for preventing ear piercing infection is the regular application of antiseptics or antibiotics [18,21,22,23,24] for a few weeks, which is not thoroughly effective as nearly 20% of all piercings lead to local infection [6]. The median duration for the ear-piercing infection treatment using antibiotic therapy was reported as 16 days by Bellaud et al. [25]. Therefore, it is imperative to develop alternative methods of prolonged and sustained piercing aftercare to prevent infection. 

Many techniques have been used to reach sustained delivery of drugs, particularly antibiotics, in wound dressing applications. Although electrospinning of medicated nanofibers has been widely used recently to fabricate wound dressings [26,27,28], the prolonged release of drugs from nanofibers remains a challenge. Yank et al. developed an electrospun wound dressing [29]; however, 90% of the loaded antibiotic was released during the first 30 min of in vitro study. In another effort, electrospun drug loaded polylactic acid (PLA) scaffolds were used to achieve sustained drug release for wound dressing applications [30]. However, the drug was released mainly in the first 72 h. Kim et al. enhanced the release of an antibiotic drug (cefoxitin sodium) out of electrospun PLGA scaffolds [31]. The efficacy of the setup was proven against *S. aureus*. However, the release of the drug reached up to one week with a rapid decrease in the zone of inhibition in the first two-hours for drug loaded scaffolds. Other fabrication methods have also faced similar challenges regarding the prolonged release. An emulsion solvent diffusion method was used to develop a drug loaded emugel microsponge to release mupirocin against *S. aureus* [32]. However, the release was sustained only for 24 h. In another study, the spray drying technique was used to develop microparticles for controlling the delivery of mupirocin calcium [33]. However, over 80% of the loaded drug was released during the first 72 h of the dissolution test. A different group reported a polyglyconate mesh dip coated in gentamicin loaded poly-lactic-*co*-glycolic acid (PLGA) to develop a wound dressing [34]. The inhibition of bacterial growth of the gentamicin loaded wound dressings maintained over two weeks. However, dip coating has limitations in terms of controlling the amount of the incorporated drug as well as thickness and geometry of the layers. Although great strides have been achieved in the field of active drug-eluting wound dressings, the release kinetics of drugs and bioactive compounds must be improved in terms of sustained and prolonged release to be used for piercing aftercare applications. 

Three-dimensional (3D) printing has been widely used to fabricate patient-tailored drug-eluting systems to achieve arbitrary geometries and hence release profiles [35,36,37,38]. However, the application of 3D printing is limited in drug-eluting wound dressings [39,40,41,42,43]. Conventional high-temperature 3D printing techniques cannot be implemented as many of the active anti-infective compounds are heat- labile [44]. Furthermore, although 3D printed wound dressings have shown improved drug-eluting characteristics, the release of the drugs has been limited to a few hours or days [40]. Therefore, there is a need to develop alternative low-temperature 3D printing methods for the fabrication of constructs that provide a prolonged drug release for piercing infection prevention.

In this study, a drug-eluting bio-absorbable scaffold was developed and is intended to cover piercing studs. This “biopierce” will stay in human tissue following piercing for up to two weeks and to prevent the development of a wound infection. A novel, low-temperature 3D printing process was developed for fabrication of the PLGA biopierces, and the organic solvent (methyl ethyl ketone (MEK)) was successfully removed after printing without affecting the drug integrity (mupirocin). The amount of the release drug over time is quantified, and the efficacy of the printed biopierces against *S. aureus* is characterized through the measurement of the zone of inhibitions in bacterial tests. The release profile of varying grades (lactic to glycolic ratios) of PLGA and concentration of mupirocin is characterized over two weeks.

## 2. Materials and Methods

### 2.1. Preparation of Biomaterial Inks

PLGA, a bio-absorbable polymer suitable for wound dressing and drug delivery applications [34,45,46], was selected as the polymeric matrix (PolySciTech, West Lafayette, IN, USA). Mupirocin, which is effective against *S. aureus* and *S. pyogenes* (Lancefield Group A), was selected as the antibacterial agent in this study [47,48]. Mupirocin (AM26100, Biosynth Carbosynth, Compton, UK) was dissolved in MEK (Sigma Aldrich, St. Louis, MO, USA) and was stirred at 200 rpm at room temperature for one hour. Biomaterial inks were prepared by adding different concentrations of the mupirocin solution to PLGA (as shown in Table 1). PLGA concentration in the biomaterial ink was set at 80% (*w*/*v*) to maintain the optimum printable biomaterial ink concentration as described in our previous study [49]. The biomaterial inks were left for overnight magnetic stirring at 200 rpm at room temperature to yield homogeneous biomaterial inks.

### 2.2. 3D Printing

**Figure 1:**
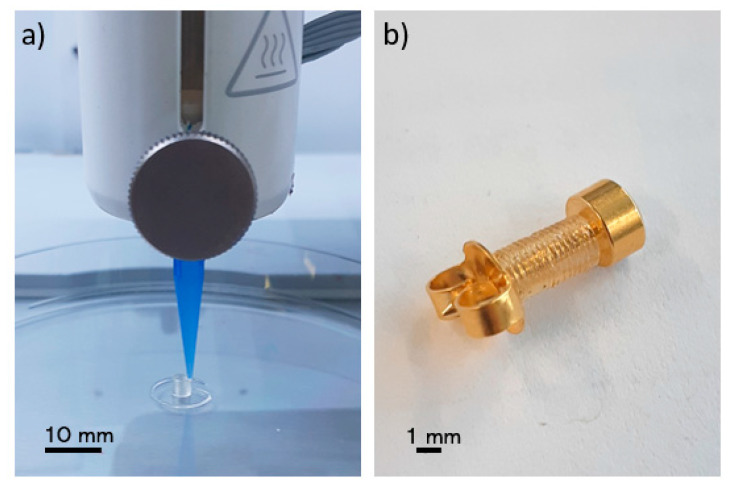
(**a**) 3D printing of a biopierce, (**b**) a biopierce assembled on a piercing stud.

A commercial 3D bioprinter (BioX, Cellink, Gothenburg, Sweden) with a pneumatic printhead (20340, Cellink, Gothenburg, Sweden) was used to print the biopierces as shown in Figure 1. A 3-mL cartridge (CSC010311101, Cellink, Gothenburg, Sweden) was filled with the biomaterial ink and was left in an inverse position to remove air bubbles. An 8.00-mm hollow cylinder with 1.60 mm inner diameter and 2.00 mm outer diameter was designed using a computer-aided design (CAD) software (SolidWorks, Waltham, MA, USA). G-codes were generated with a slicer software (Slic3r, Repetier-Host, Willich, Germany) in such a way as to have only a single thread of the filament in each layer. Biopierces were printed with a 410 μm conical nozzle (NZ3220005001, Cellink, Gothenburg, Sweden) (Figure 1a). A printed biopierce fitted on a piercing stud (R993-S, Studex, Gardena, CA, USA) is shown in Figure 1b. The printing pressure was 550 kPa, and the printing speed was set at 1.00 mm/s to minimize any deformation of the printed construct while printing. A 10.00-mm length scaffold was printed for bacterial testing.

### 2.3. Post-Printing Requirements

Solvent removal: Proton nuclear magnetic resonance (^1^H NMR) spectroscopy was used to confirm the complete removal of the solvent. Considering the vapor pressure of MEK at 20 °C (9.5 kPa), similar to our previous study [49], the printed scaffolds were dried in a vacuum flask connected to a vacuum line of a fume hood for one week. A scaffold was placed in 1 mL of deuterium oxide (D_2_O) 99.9% (Cambridge Isotope Laboratories Inc., Tewksbury, MA, USA) to leach any remaining solvent into D_2_O overnight. The trace amount of MEK was detected by ^1^H NMR spectroscopy (300 MHz, Bruker, MA, USA) with 16 scans setting [49].

Drug integrity: The integrity of mupirocin after solvent removal and over the release duration was also investigated. Mupirocin contains structurally sensitive moieties, such as epoxide and ester, which upon hydrolysis could either alter or diminish its antibacterial properties. Liquid chromatography high-resolution mass spectrometry (LC-HRMS) was run for sample integrity analysis and was conducted as follows: Thermo Accela UHPLC Pump, Thermo Exactive HRMS fitted with an ESI source and Thermo PDA. A Kinetex core–shell 100 Å C18 column (2.1 × 50 mm, 1.7 μm, Phenomenex) was used with a mobile-phase flow rate of 0.5 mL/min and injection volume of 10 μL (all samples were prepared in CH_3_OH). The following elution method was used (A = H_2_O (0.1% formic acid), B = CH_3_CN (0.1% formic acid)): 5% B from 0.0 to 0.2 min, linear gradient from 5% B at 0.2 min to 99% B at 4.8 min, 99% B from 4.8 to 8.0 min, linear gradient from 99% B at 8.0 min to 5% B at 8.5 min, and 5% B from 8.5 to 10.0 min. The following HRMS parameters were used: positive ionization mode, mass resolution of 30,000, mass range of *m*/*z* 190 to 2000, spray voltage of 2.0 kV, the capillary temperature of 300 °C, S-lens RF voltage of 60.0%, maximum injection time of 10 ms, and 1 microscan. The system was controlled by Thermo Xcalibur software modules. 

### 2.4. In Vitro Drug Release Study

Phosphate buffered saline (PBS) (VWRL0119, VWR, Radnor, PA, USA) was used as the dissolution medium (pH = 7.4). To appropriately simulate in vivo drug release conditions [40], the scaffolds were incubated in 4 mL of PBS at 37 °C, and the medium was stirred at 200 rpm. To eliminate the effect of the medium removal on the drug release kinetics at each time point, the scaffolds were incubated 1 day, 2 days, 1 week, and 2 weeks prior to the tests. The scaffolds were taken out at the time points for the scaffold diffusion assay, and the dissolution medium was used in the in vitro drug release study and the disk diffusion assay.

The amount of released mupirocin from the scaffold over time was quantified via LC-HRMS. Each sample, in triplicate, was diluted by a half to ensure limit of detection was not reached prematurely. Peak areas were calculated from the extracted ion count of the released mupirocin, and a calibration curve was used to determine the total release. To develop the calibration curve, all parameters were maintained similar to the drug integrity study and the following concentrations were injected: 1.56, 3.13, 6.25, 12.50, 25.00, 50.00, 75.00, and 100.00 µg/mL. The calibration curve was produced using the extracted ion count peak area for mupirocin.

### 2.5. In Vitro Mupirocin Efficacy

The European Committee on Antimicrobial Susceptibility Testing (EUCAST) reports the mupirocin minimum inhibitory concentration (MIC) of 1 mg/L for *S. aureus* [50]. The efficacy of biopierces against *S. aureus* was studied in terms of scaffold and disk diffusion assays by characterizing the zone of inhibition of the eluted mupirocin. *S. aureus* ATCC^®^ 25923™ (Oxoid, Nepean, ON, Canada) is the recommended quality control strain for disk diffusion testing following the Clinical and Laboratory Standards Institute (CLSI) standards [51]. The isolate was grown from frozen stock and subcultured twice onto Columbia agar (Oxoid Columbia Blood Agar Base # CM0331) supplemented with 5% defibrinated sheep blood (SBA) (Quad Five Donor Sheep Blood Defibrinated # 610-500) before testing. Colonies were picked from 18 h growth on SBA using a cotton swab, re-suspended in tryptic soy broth (TSB) (BD Bacto Tryptic Soy Broth # 211825), and adjusted to 1.5 × 10^8^ CFU/mL by visual comparison with a 0.5 McFarland standard. A bacterial lawn was inoculated on to cation-adjusted Mueller-Hinton agar plates (MHA) (BD BBL Mueller Hinton II Agar # 211438). All media were prepared by Atlantic Veterinary College (AVC) Central Services media preparation laboratory following manufacturers’ guidelines. 

Scaffold diffusion assay: Printed scaffolds were placed onto inoculated MHA plates within 15 min of inoculation. The agar was sliced with sterile sharp tweezers and the scaffold embedded into the agar. MHA plates were incubated at 35 ± 2 °C in ambient air for 24 h. Images were taken of the agar plates and were processed by Fiji image processing software (ImageJ 1.53c, GNU General Public License, Bethesda, Rockville, MD, USA) [52] to determine the zone of inhibition. The images were converted to 8-bit images, and the threshold was set between 151 and 170 to specify the zone of inhibition. The pixel size was 20 µm, and the uncertainty in the measurement of the zone of inhibition areas was 0.1%. To eliminate the effect of the size of the scaffolds, the area of the corrected zone of inhibition, simply called zone of inhibition, was calculated by deducting the scaffold area out of the zone of inhibition area. A drug-free scaffold was used as a negative control. All tests were implemented in triplicate and mean values and standard deviations were reported. 

Disk diffusion assay: Sterile paper disks were moistened using 30 µL of the dissolution medium (2017-006, Whatman, Marlborough, UIK, GE Health Sciences, Chicago, IL, USA). The disks were dried at room temperature and were placed on MHA plates within 15 min of inoculation. MHA plates were incubated at 35 ± 2 °C in ambient air for 24 h [53]. Images of agar plates were taken and were processed similar to the scaffold diffusion assay. The area of the corrected zone of inhibition, simply called zone of inhibition, was calculated by deducting the disk area out of the zone of inhibition area [34]. A drug-free disk in the medium was used as a negative control, and a 200-µg mupirocin antimicrobial susceptibility disk (CT0523B, Oxoid, Hampton, VA, USA) was used as a positive control. All tests were implemented in triplicate and mean values and standard deviations were reported.

## 3. Results and Discussion

### 3.1. Solvent Removal

**Figure 2:**
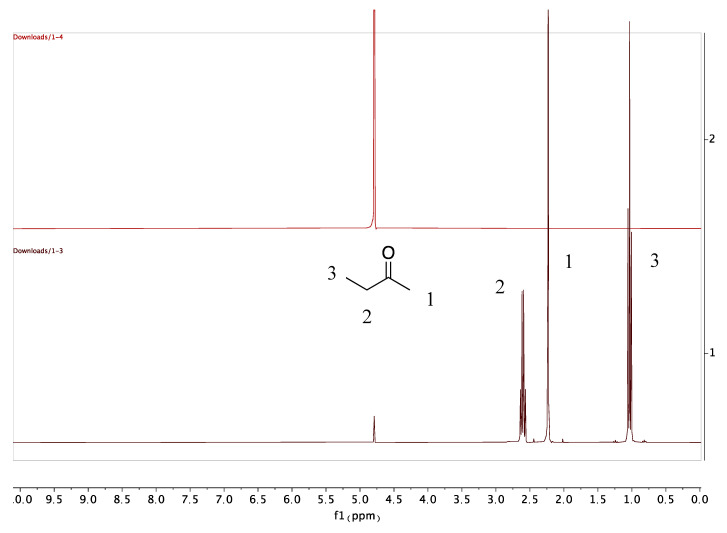
Stacked ^1^H NMR spectra showing the complete removal of MEK after drying (red) and residual MEK before drying (black). MEK ^1^H NMR (400 MHz, Methanol-d_4_) δ 1 = 3H, CH_3_, s. 2 = 2H, CH_2_, q. 3 = 3H, CH_3_, t. D_2_O signal referenced to 4.790 ppm.

The residual solvent (MEK) peaks in PLGA scaffolds were detected immediately after 3D printing. However, no MEK peak was found after a week of vacuum drying of the scaffolds (Figure 2). The results showed that vacuum drying of the scaffolds is an effective strategy to remove the residual solvent. The drying time can be further decreased using a lower vacuum pressure. However, a lower vacuum pressure increases the risk of bubble formation in the scaffold.

### 3.2. Drug Integrity Study

**Figure 3:**
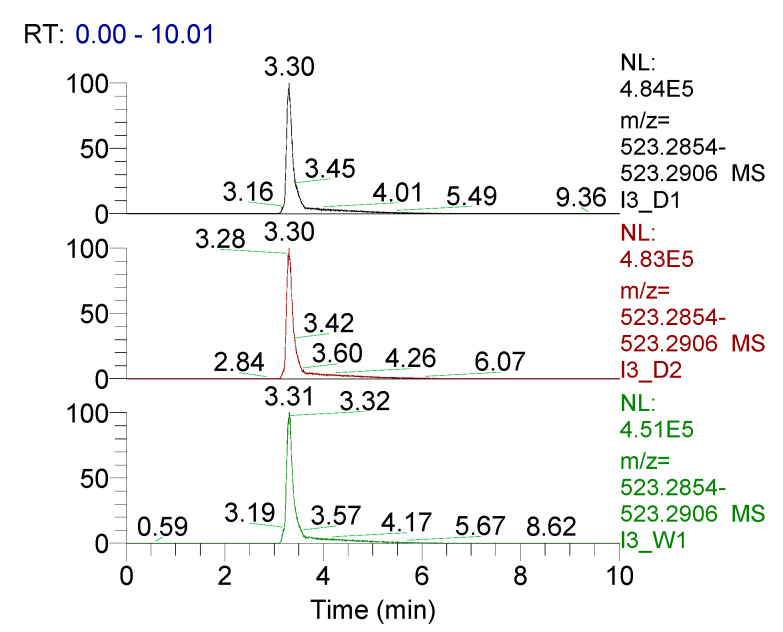

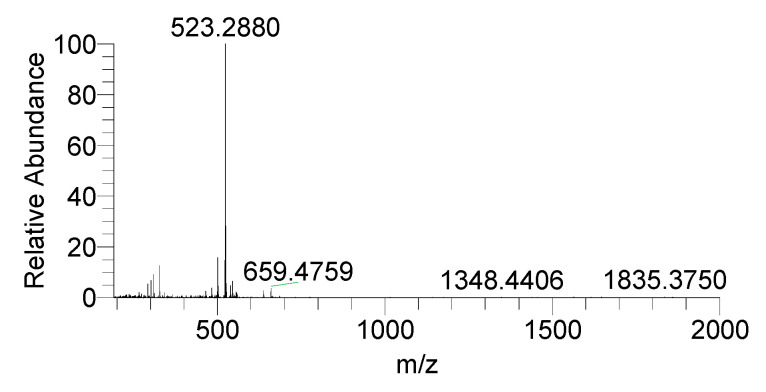
LC-HRMS data of mupirocin elution after day 1 (black), day 2 (red) and week 1 (green), along with accurate mass 523.2880 *m/z* [M+Na]^+^.

Using LC-HRMS for analysis, samples of dissolution medium were taken at day 1, day 2, and week 1 to determine if the structural integrity of mupirocin was maintained (Figure 3). Over a week, the retention time of mupirocin was unchanged, along with the absence of an appearance of any degradation products. These data, combined with accurate mass (calculated 523.2872 *m*/*z*, observed 523.2880 *m*/*z* [M+Na]^+^) provide clear evidence that the integrity of mupirocin is uncompromised during this process.

### 3.3. Zone of Inhibition Characterization 

**Figure 4:**
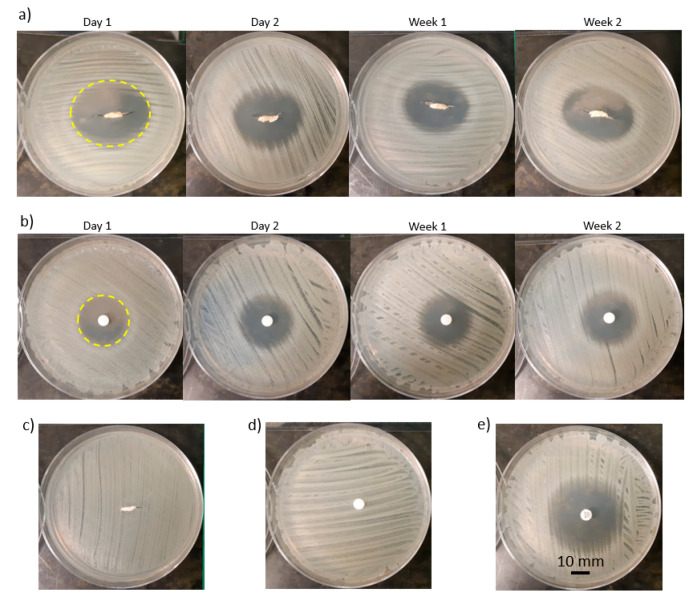
Antimicrobial sensitivity testing: (**a**) scaffold diffusion assays and (**b**) disk diffusion assays of LG5050-20 over four time points and determining the zone of inhibition (Day 1), (**c**) a negative control of the scaffold diffusion assays, (**d**) a negative control of the disk diffusion assays, (**e**) a positive control of the disk diffusion assays.

As an example of the bacterial tests, the zone of inhibition for LG5050-20 over four time points for the scaffold and disk diffusion assays are shown in Figure 4a,b. The zone of inhibition of the scaffolds was elliptical-shape (Figure 4a—Day 1) while the zone of inhibition of the disks was circle-shape (Figure 4b—Day 1), which reflects the respective geometries of the scaffolds and disks. The elliptical shape of the scaffolds conforms with the zone of inhibition shape of a previously studied gentamicin loaded PLA catheter [54]. Two negative controls were the performed, a drug free scaffold (Figure 4c) and the dissolution medium (Figure 4d), ensuring all antibacterial activity was due to mupirocin. Finally, a positive control, 200 µg loaded mupirocin disks were used as a direct comparison (Figure 4e).

### 3.4. Effects of Drug Concentration on Drug Release

**Figure 5:**
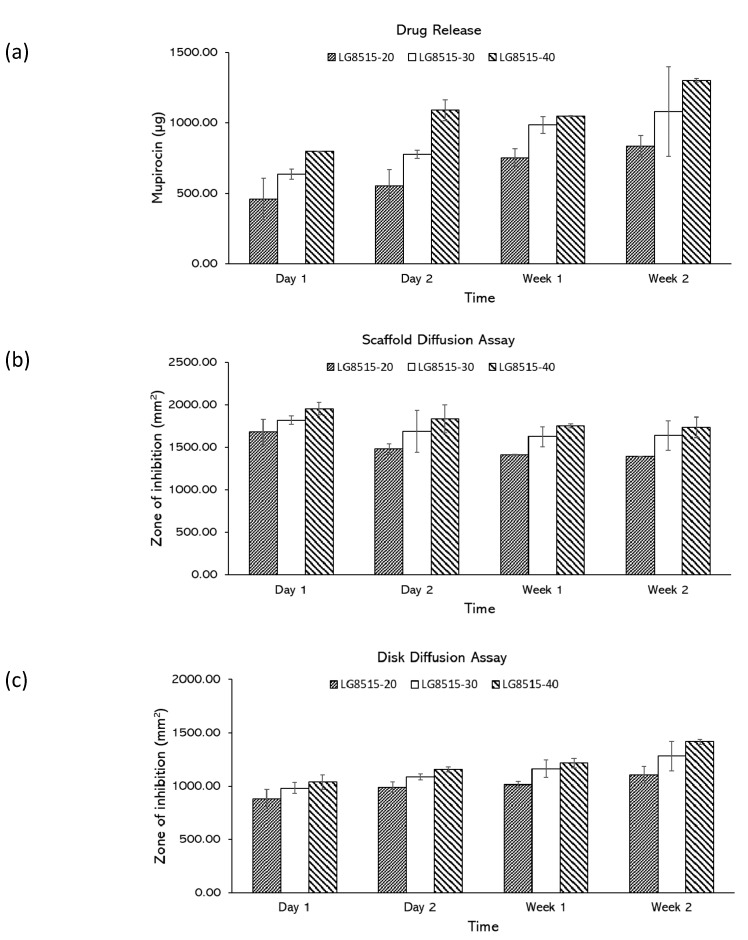
(**a**) amount of the released drug, (**b**) zone of inhibition in scaffold diffusion assays, (**c**) zone of inhibition in disk diffusion assays. Error bars represent standard deviations.

The effect of drug concentration on the drug release was investigated. According to the drug release study (Figure 5a), mupirocin was successfully released over two weeks for all concentrations, and the amount of the released drug increased over time. The amount of the released mupirocin from LG8515-20, LG8515-30, and LG8515-40 to the media increased from 459.83 ± 146.31 to 835.20 ± 76.17 µg, 636.39 ± 35.51 to 1080.83 ± 318.22 µg, 797.69 ± 1.62 to 1303.04 ± 11.73 µg, respectively, over two weeks. A higher concentration of drugs in the scaffolds led to a higher amount of the released drug at each time point. The effect of drug concentration on the drug release was further examined by scaffold and disk diffusion assays.

Scaffold diffusion assay: The scaffolds with a higher concentration of the drug had a larger zone of inhibition, as shown in Figure 5b. Increasing the amount of mupirocin increased the zone of inhibition, however, the increase is not linearly dependent on the drug concentration. For instance, doubling the drug concentration in LG8515-20 did not double the zone of inhibition. The zone of inhibition values of LG5050-20 and LG5050-40 at day 1 were 1681.82 ± 150.16 mm^2^ and 1953.74 ± 75.52 mm^2^, respectively. The observed trend is comparable with the developed polyglyconate dressing coated with gentamicin loaded PLGA [34]. The zone of inhibition was increased by increasing the drug concentration [34].

The zone of inhibition of LG8515-20, LG8515-30, and LG8515-40 gradually decreased over time, which indicates that the drug release rate of the scaffolds is reduced over time. The zone of inhibition of LG8515-20, LG8515-30, and LG8515-40 decreased from 1681.82 ± 150.16 to 1389.94 ± 0.51 mm^2^, 1818.79 ± 48.27 to 1637.37 ± 173.77 mm^2^, and 1953.74 ± 75.52 to 1732.48 ± 119.61 mm^2^, respectively over two weeks.

Disk diffusion assay: The zone of inhibition for the disk diffusion assay attributed to the previously studied scaffolds are shown in Figure 5c. Increasing the drug concentration increased the zone of inhibition at each time point. The zone of inhibition of LG8515-20, LG8515-30, and LG8515-40 increased from 879.70 ± 92.61 to 1108.23 ± 78.24 mm^2^, 983.18 ± 48.94 to 1282.46 ± 137.90 mm^2^, and 1038.63 ± 66.03 to 1418.19 ± 21.28 mm^2^, respectively, over two weeks. The increase in the zone of inhibition for disks is consistent with the decrease in the zone of inhibition area for the scaffolds over time. More release into the medium (as represented by disk diffusion assays) results in less release from the scaffold over time. The results of the disk diffusion assay conform with the drug release test results.

### 3.5. Effects of PLGA Composition on Drug Release

**Figure 6:**
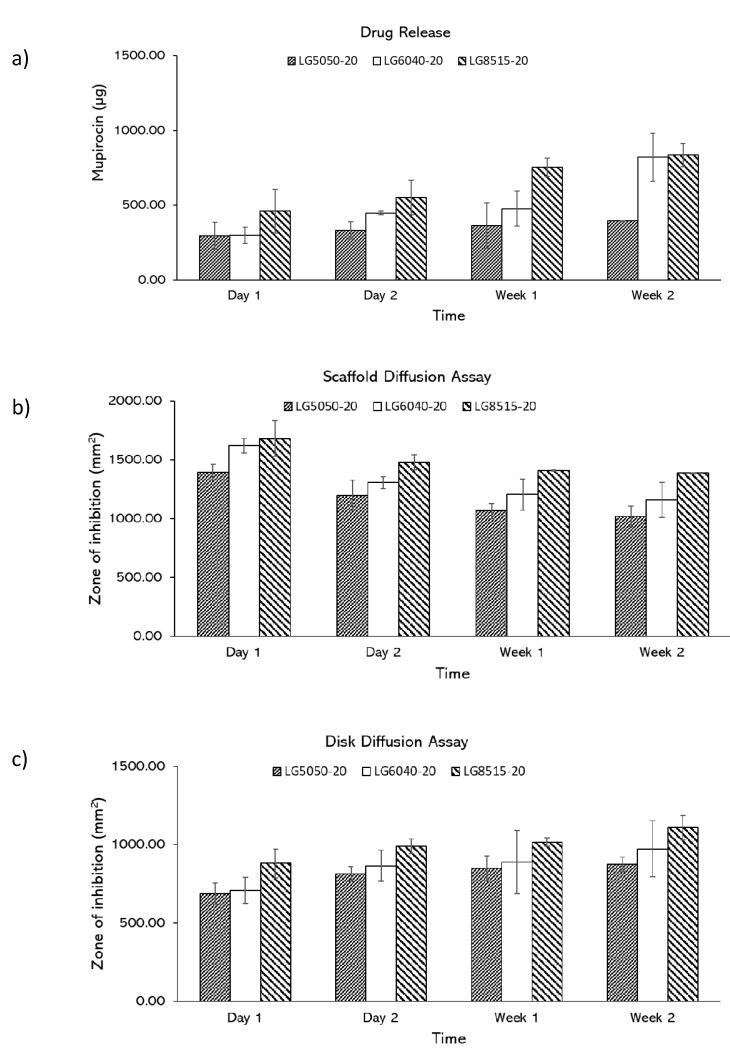
(**a**) amount of the released drug, (**b**) zone of inhibition in scaffold diffusion assays, (**c**) zone of inhibition in disk diffusion assays. Error bars represent standard deviations.

The effect of PLGA composition on the drug release was investigated. In drug release study, mupirocin was shown to be released over two weeks, and the amount of the released drug increased over time (Figure 6a). The amount of the released mupirocin from LG5050-20, LG6040-20, and LG8515-20 to the media increased from 294.28 ± 90.27 to 394.62 ± 0.88 µg, 298.46 ± 52.89 to 821.08 ± 160.11 µg, and 459.83 ± 146.31 to 835.20 ± 76.17 µg, respectively over two weeks. Higher lactic ratio in PLGA scaffolds led to higher amount of the released drug at each time point. The effect of PLGA composition on the drug release was further examined by scaffold and disk diffusion assays. 

Scaffold diffusion assay: As shown in Figure 6b, the zone of inhibition of LG5050-20, LG6040-20, and LG8515-20 scaffolds gradually decreased, which indicates that the drug release rate of the scaffolds is reduced over time. The zone of inhibition of LG5050-20, LG6040-20, and LG8515-20 scaffolds decreased from 1395.19 ± 66.19 to 1018.49 ± 85.52 mm^2^, 1620.97 ± 63.57 to 1160.49 ± 150.11 mm^2^, and 1681.82 ± 150.16 to 1389.95 ± 0.51 mm^2^, respectively, over two weeks. In addition, the amount of the released drug at each time point increased per increase of the lactic to glycolic ratio in PLGA. The degradation rate of PLGA scaffolds increases with an increase in glycolic ratios [55]; however, in this study, higher lactic ratio PLGA scaffolds released more drugs. Therefore, it is concluded that at least for the first 14 days of the drug release, the release is driven mostly by diffusion. This agrees with Chew et al. that reported an acceleration in the drug release of PLGA 50:50 after 14 days [56], which was believed to be due to the change in release mechanism from diffusion to degradation. Additionally, the increase in release from higher L:G ratio can be due to the higher solubility of higher ratio PLGA in MEK [57].

Disk diffusion assay: The zone of inhibition for the disk diffusion assay attributed to the previously studied scaffolds are shown in Figure 6c. The zone of inhibition for each PLGA composition increased over time, which indicates that the amount of the released drug into the medium was increased. The inhibition zone of LG5050-20, LG6040-20, and LG8515-20 increased from 686.88 ± 68.92 to 873.87 ± 46.12 mm^2^, 708.40 ± 82.04 to 972.93 ± 178.88 mm^2^, and 879.70 ± 92.61 to 1108.23 ± 78.24 mm^2^, respectively, over two weeks. These results agree with the corresponding scaffold diffusion study: a decrease in the zone of inhibition of the scaffolds and an increase in the zone of inhibition of the disks over time. The results of the disk diffusion assay conform with the drug release test results. In addition, the disk diffusion assay results conform with drug release study results. 

### 3.6. Release Potential (RP) and Total Zone of Inhibition

Further investigation of the results showed that the zone of inhibition of the scaffolds and disks did not change with the same rate over time. Release potential (RP) was defined as the ratio of the scaffold zone of inhibition over the disk zone of inhibition, which expresses the potential of a scaffold to release the drug. It is expected that the release potential of the scaffolds declines over time and reaches its limit of zero by full degradation of the scaffold. 

**Figure 7:**
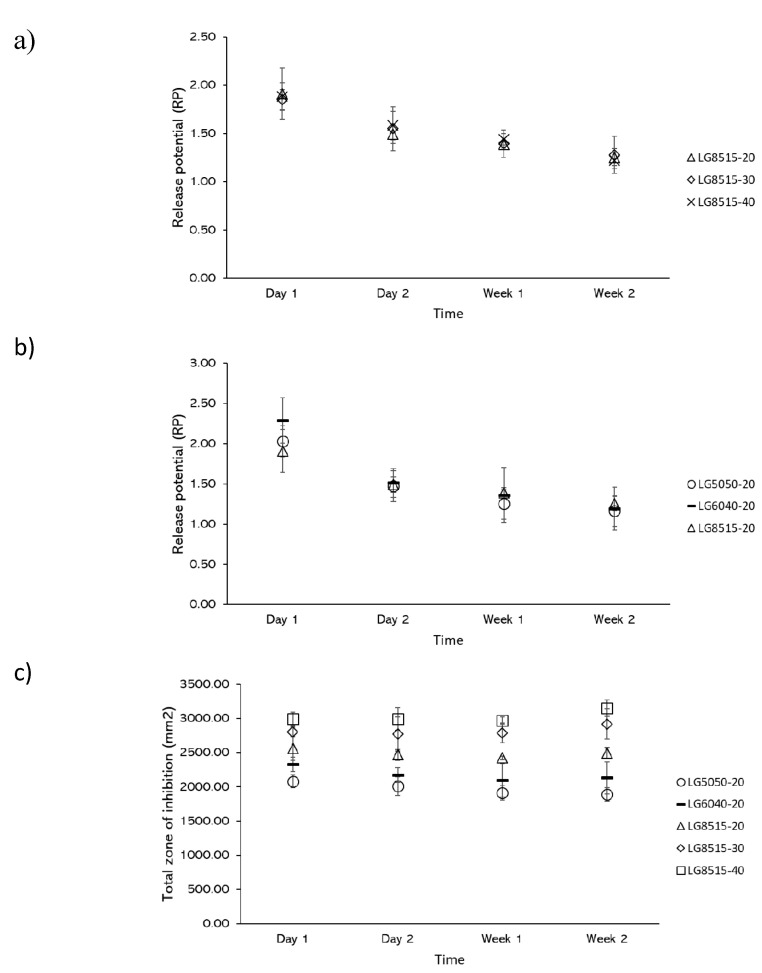
Release potential (RP) and the effect of (**a**) mupirocin concentration and (**b**) PLGA composition on the release potential (RP), (**c**) total zone of inhibition.

The release potential for varying mupirocin concentrations and PLGA grades at each time point is shown in Figure 7a,b, respectively. From these results, it can be concluded that the release potential is independent of the PLGA grade or concentration of mupirocin. This highlights an interesting point as the release potential can be used for the design of mupirocin-releasing PLGA constructs.

The total zone of inhibition that is defined as the summation of the zone of inhibition of a scaffold and the corresponding disk at each time point is used to further investigate the drug release. As shown in Figure 7c, the total zone of inhibition for each scaffold remained consistent over time; the average total zone of inhibition for LG5050-20, LG6040-20, LG8515-20, LG8515-30, and LG8515-40 was 1974.56 ± 219.84 mm^2^, 2182.03 ± 369.13 mm^2^, 2488.35 ± 210.60 mm^2^, 2820.98 ± 368.47 mm^2^, and 3026.84 ± 234.53 mm^2^, respectively. Total zone of inhibition increased by increasing the lactic to glycolic ratio of PLGA: LG8515-20 > LG6040-20 > LG5050-20. In addition, total zone of inhibition increased by increasing the drug concentration: LG8515-40 > LG8515-30 > LG8515-20. Therefore, for achieving the highest efficacy, the use of a higher grade of lactic to glycolic ratio is recommended.

### 3.7. Mechanical Integrity

**Figure 8:**
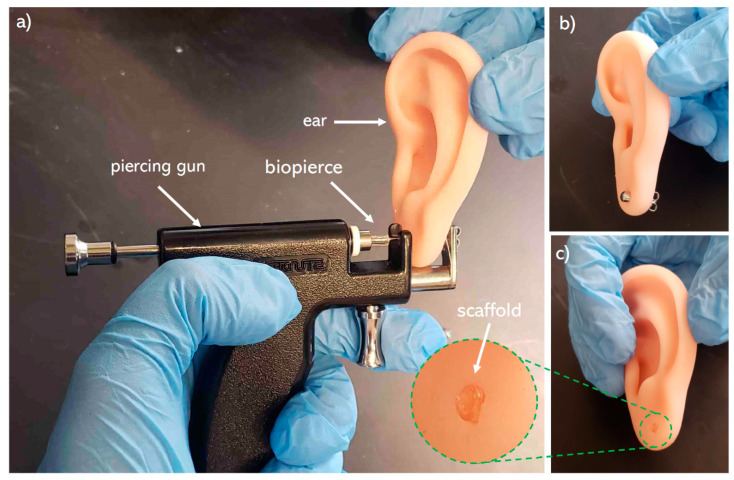
(**a**) piercing of a plastic ear model with a piercing stud covered by a biopierce, (**b**) a model ear after being pierced by biopierce covered piercing stud, (**c**) biopierce left in the ear model lobe after removing the piercing stud.

Mechanical integrity of the biopierces are examined through a piercing test. As shown in Figure 8, the biopierce maintains its integrity during and after piercing. In addition, the biopierce maintains its shape and integrity within the punctured tissue after removing the piercing stud.

## 4. Conclusions

A novel drug-eluting bio-absorbable scaffold called biopierce, which is intended to cover piercing studs and prevent piercing infection, was introduced and characterized. In terms of the biomaterial ink composition, PLGA, mupirocin, and MEK were used as the biopolymer, antimicrobial agent, and solvent, respectively. A low-temperature 3D printing technique was used to print the biopierces, and ^1^H NMR spectroscopy was used after the vacuum drying of the scaffold to confirm the complete removal of MEK. Scaffolds in different PLGA composition and loaded mupirocin amounts were examined to characterize the release amounts of mupirocin. LC-HRMS was used to confirm the structural integrity of mupirocin and to quantify the amount of the released drug over time. In addition, the effective release of mupirocin against *S. aureus*, indicated as the zone of inhibition, was studied in the scaffold and the disk diffusion assays, respectively. The drug release of scaffolds decreased over time while the drug concentration in the dissolution medium increased. Increasing mupirocin concentration and lactic to glycolic ratio of PLGA increased the zone of inhibition in the scaffold and disk diffusion assays. PLGA 85:15 is recommended to be used as the polymeric matrix because of its superior drug release and printability characteristics.

The zone of inhibition was consistently maintained over 14 days that showed the efficacy of biopierces. The outcomes of this research can be used for tissue piercing applications as well as other wound dressing research. For further development of this research, incorporating analgesic and anti-proliferative agents is recommended. More importantly, the antimicrobial properties of mupirocin in the presence of other drugs must be investigated. Overall, this work is an exciting area of development that can lead to the next generation of drug-eluting wound dressings.

## Figures and Tables

**Table 1:** Biomaterial ink composition in terms of lactic (L) to glycolic (G) ratio and molecular weight of PLGA and concentration of mupirocin.

Biomaterial Ink	L:G	Molecular Weight (kDa)	Mupirocin in PLGA (% *w*/*w*)
LG5050-20	50:50	85–100	20
LG6040-20	60:40	100–200	20
LG8515-20	85:15	100–200	20
LG8515-30	85:15	100–200	30
LG8515-40	85:15	100–200	40
